# *OSskcm*: an online survival analysis webserver for skin cutaneous melanoma based on 1085 transcriptomic profiles

**DOI:** 10.1186/s12935-020-01262-3

**Published:** 2020-05-19

**Authors:** Lu Zhang, Qiang Wang, Lijie Wang, Longxiang Xie, Yang An, Guosen Zhang, Wan Zhu, Yongqiang Li, Zhihui Liu, Xiaochen Zhang, Panpan Tang, Xiaozheng Huo, Xiangqian Guo

**Affiliations:** 1 Department of Preventive Medicine, Institute of Biomedical Informatics, Bioinformatics Center, School of Software, School of Basic Medical Sciences, Henan University, Kaifeng, 475004 Henan China; 2 Henan Provincial Engineering Centre for Tumor Molecular Medicine, Henan University, Kaifeng, 475004 Henan China; 3 Department of Anesthesia, Stanford University, Stanford, CA 94305 USA

**Keywords:** Cutaneous melanoma, Survival, Prognosis, Biomarker

## Abstract

**Background:**

Cutaneous melanoma is one of the most aggressive and lethal skin cancers. It is greatly important to identify prognostic biomarkers to guide the clinical management. However, it is technically challenging for untrained researchers to process high dimensional profiling data and identify potential prognostic genes in profiling datasets.

**Methods:**

In this study, we developed a webserver to analyze the prognostic values of genes in cutaneous melanoma using data from TCGA and GEO databases. The webserver is named Online consensus Survival webserver for Skin Cutaneous Melanoma (*OSskcm*) which includes 1085 clinical melanoma samples. The *OSskcm* is hosted in a windows tomcat server. Server-side scripts were developed in Java script. The database system is managed by a SQL Server, which integrates gene expression data and clinical data. The Kaplan–Meier (KM) survival curves, Hazard ratio (HR) and 95% confidence interval (95%CI) were calculated in a univariate Cox regression analysis.

**Results:**

In *OSskcm*, by inputting official gene symbol and selecting proper options, users could obtain KM survival plot with log-rank *P* value and HR on the output web page. In addition, clinical characters including race, stage, gender, age and type of therapy could also be included in the prognosis analysis as confounding factors to constrain the analysis in a subgroup of melanoma patients.

**Conclusion:**

The *OSskcm* is highly valuable for biologists and clinicians to perform the assessment and validation of new or interested prognostic biomarkers for melanoma. *OSskcm* can be accessed online at: http://bioinfo.henu.edu.cn/Melanoma/MelanomaList.jsp.

## Background

Cutaneous melanoma (CM) is one of the most lethal malignancies of skin [1]. It was estimated that 287,700 new cases of melanoma and 60,700 deaths of melanomas occurred worldwide in 2018 [2]. Patients with metastatic melanoma have a shorter long-term survival time. Moreover, survival outcomes can vary widely among patients even within the same stage due to the biological heterogeneity of melanoma. At present, the methods commonly used in the treatment of melanoma include surgical resection, chemotherapy and immunotherapy. Only a few patients with advanced melanoma have a persistent response to surgical resection and chemotherapy. Some researchers have used mouse models to analyze the causes of drug resistance, possibly due to changes in metabolic levels in the state of obesity [3, 4]. Weight control can improve the effectiveness of medications and help reduce melanoma metastasis [5]. In addition, the combination of chemotherapy drugs may improve drug resistance [6, 7]. However, because of the molecular heterogeneity, not all the melanoma patients responded well to the treatments. Mutant BRAF has been shown to be significantly associated with worsen overall survival and metastasis free survival of melanoma [8], meanwhile mutant BRAF has been also proven to be a good therapeutic target for melanoma, but the resistance of small molecule drugs against mutant BRAF for melanoma is invariably observed [9]. Therefore, it is imperative to develop novel prognostic biomarkers for risk stratification and treatment optimization in melanoma patients. The specific and novel biomarker may provide the opportunities for guidance of personalized therapeutic interventions and new therapeutic target development.

High-throughput RNA-sequencing (RNA-Seq) has been shown to successfully measure gene expression, discover novel transcripts and identify differentially expressed genes [10]. BRAF and NRAS mutations have been used as molecular biomarkers in evaluating the clinical course of melanoma. Identification of novel molecular biomarkers becomes an area of interests to clinicians and researchers. Ideally, prognostic biomarkers are sensitive, specific, reliable, rapidly analyzable and cost effective. To date, a number of prognostic biomarkers have been proposed in melanoma [11]; however, most of these putative biomarkers lack independent validation in multiple cohorts. Mining available transcriptome data with appropriate clinical follow-up information offers opportunities to prescreen and validate new prognostic biomarkers [12]. Currently, there are several web-browsers, such as PRECOG [13], KM plotter [14] and CaPSSA [15], which have provided survival analysis based on gene expression. However, most of these prognostic analysis web servers only provide data from TCGA, without data from other sources such as GEO and published literatures. As we all know, the most important and difficult part of the biomarker development is to validate the performance of potential biomarker in multiple independent datasets, in this current study, we developed an Online consensus Survival webserver for Skin Cutaneous Melanoma, named *OSskcm*, which analyzes tumor gene expression profiles and clinical follow-up information of 1085 melanoma patients from multiple independent cohorts. The *OSskcm* webserver is registration-free and can assist biologists and clinicians to evaluate the prognostic potency of genes of interests and identify potential therapeutic targets.

## Materials and methods

Expression profiling and clinical follow-up data used in *OSskcm* were collected from Gene Expression Omnibus (GEO; https://www.ncbi.nlm.nih.gov/geo/) and The Cancer Genome Atlas (TCGA; https://cancergenome.nih.gov/) by searching with the keywords of “cutaneous melanoma” and “survival”. Only datasets containing mRNA expression profiling data, clinical survival information, and at least 20 cutaneous melanoma cases were included. The Kaplan–Meier (KM) survival curves were set up using a central server, Hazard ratio (HR) and 95% confidence interval (95%CI) were calculated in a univariate Cox regression analysis. Risk factors, including race, stage, gender, age and type of therapy, can be selected for a subgroup analysis. The *OSskcm* is hosted in a windows tomcat server. Server-side scripts were developed in Java script, which control the request of analysis and return the analysis results. The database system is managed by a SQL Server, which integrates gene expression data and clinical data. The central server for *OSskcm* can be accessed at http://bioinfo.henu.edu.cn/Melanoma/MelanomaList.jsp. More details of the methods of *OSskcm* development have been described [16–19].

## Results

### Clinical characteristics of cutaneous melanoma cohorts in *OSskcm*

We collected 1085 unique patients, including 615 patients from six GEO datasets and 470 patients from TCGA dataset. These melanoma samples include 221 primary cutaneous melanomas, 851 metastatic melanomas, and the tumor origin of 13 patients was unknown. (Table 1). The median age of patients is 59 years old. 762 patients have overall survival (OS) data, and the median overall survival is 39.3 months. In addition, 475 patients have progression-free survival (PFS) data, 665 patients have disease-specific survival (DSS) data, 470 patients have progression-free interval (PFI) data, and 150 patients have distant metastasis-free survival (DMFS) data.

**Table 1:** Clinical properties of cutaneous melanoma patients in *OSskcm*

GEO ID	References	Platform	No. of samples	Death event	Median overall survival (months)	Ages (years)	Gender (male/female)	Primary/metastatic	Stage (I/II/III/IV)
GSE17275	[20]	GPL1930	60	41	64.00 (46.25–89.50)	NA	NA	20/40	2/8/19/31
GSE22155	[21]	GPL6102 GPL6947	70	60	7.27 (2.10–13.80)	56.63 ± 14.58	39/31	0/70	0/0/3/67
GSE46517	[22]	GPL96	84	40	71 (55–89)^a^	77.03 ± 26.37	39/24^c^	31/53	12/15/11/24
GSE50509	[23]	GPL10558	19	15	18.11 (8.63–26.53)	57.68 ± 15.49	12/7	0/19	NA
GSE65904	[24]	GPL10558	214	102	17.80 (7.03–41.83)^b^	62.35 ± 14.40	124/89^‡^	16/188^‡^	NA
GSE98394	[25]	GPL16791	51	18	93.50 (35.00–111.00)	NA	31/20	51/0	12/22/10/0^‡^
GSE19234	[26]	GPL570	38	24	38.08 (23.57–65.90)	62.66 ± 17.86	24/14	0/38	0/0/34/4
GSE53118	[27]	GPL6884	79	47	79.74 (28.81–120.05)	55.49 ± 15.27	50/29	0/79	0/0/79/0
TCGA	[28]	Illumina HiSeqV2	470	216	34.45 (14.90–75.17)	58.22 ± 15.73	290/180	103/364^‡^	77/140/171/23^‡^
Total			1085	563	39.30 (15.92–88.00)	59.14 ± 15.55	609/394	221/851	131/215/268/149

*NA* not available

^a^The survival endpoint was defined as event-free survival from resection until death

^b^The survival endpoint was defined as disease-specific survival

^c^Partial data missing

### The application of *OSskcm* webserver

**Fig. 1:**
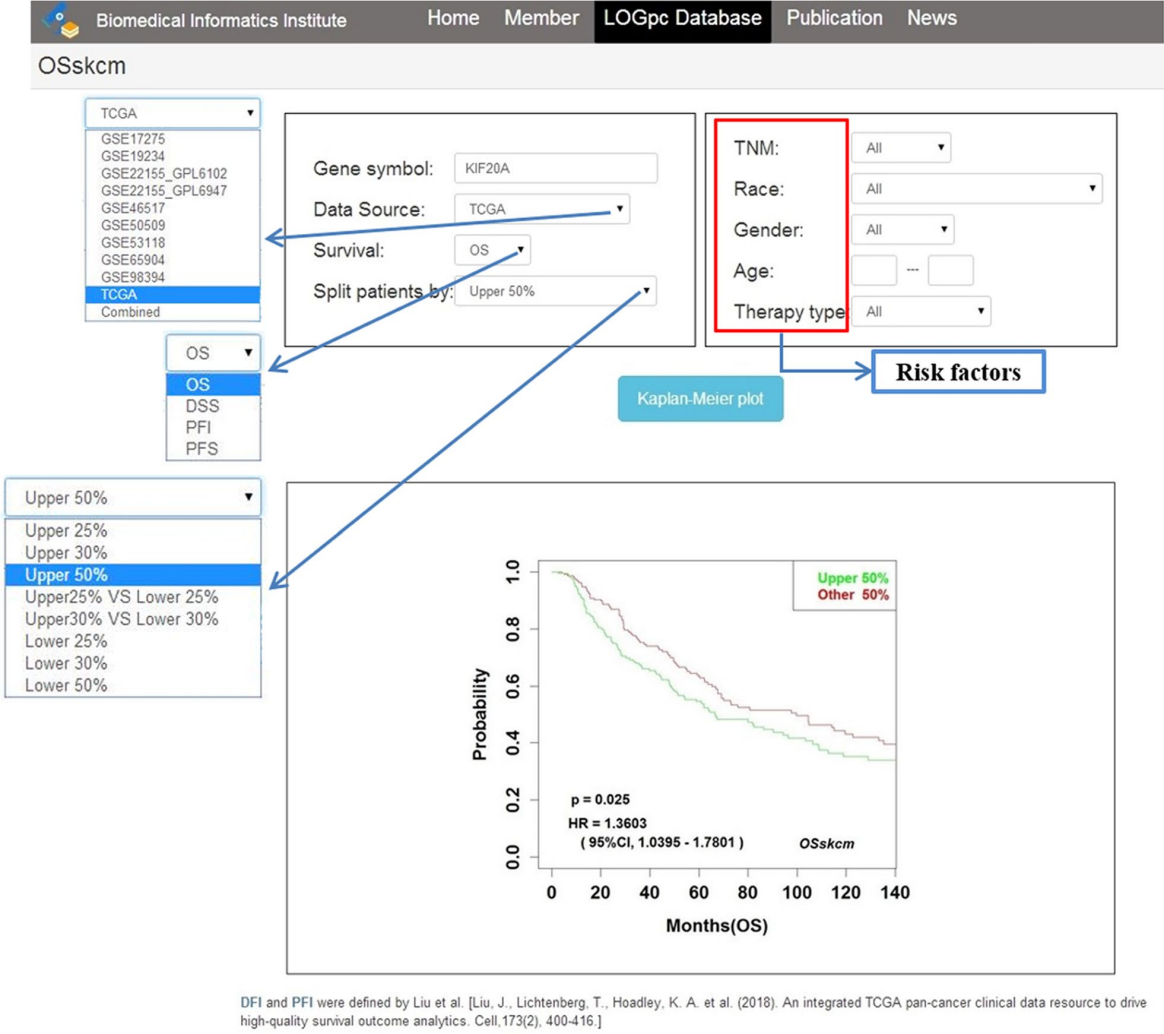
The usage and output web page of *OSskcm* webserver

To apply *OSskcm* to determine the prognostic value of gene of interest, users only need to input an official gene symbol into “Gene symbol” dialog box, and choose “Data source” as either one individual dataset or combined datasets, then select one of the “Survival” terms such as OS, PFS, DSS or PFI, and select a appropriate cut-off value of gene expression stratification by “Split patients by”. After then click the ‘Kaplan–Meier plot’ button, the KM plots with log-rank *P* value and HR with 95%CI will be shown on the output web page (Fig. 1). If users are interested in the prognostic significance of biomarkers in a particular subgroup of patients, such as races, tumor stages and treatment methods, they may select corresponding risk factors to filter the patients prior to Kaplan–Meier analysis.

### Validation of previously published cutaneous melanoma biomarkers

A PubMed search was performed using keywords of ‘cutaneous melanoma’, ‘survival’, and ‘biomarker’ to identify genes previously reported as prognostic biomarkers for cutaneous melanoma in the literatures. In total, 30 such prognostic genes were validated in *OSskcm* (listed in Table 2). These biomarker candidates were generally detected by tissue-based immunohistochemistry or immunofluorescent staining.

**Table 2:** Performance of previously published protein prognostic biomarker candidates in *OSskcm*

Gene symbol	Literature data				Validation results			
	References	*n*	Survival endpoint	Prognostic significance of high expression	*HR* (95%CI)	Log-rank *P* value	Datasets	Cut off
*KLK7*	[20]	45	OS	Good	2.65 (1.27–5.53)^†^	0.0095	GSE17275	Upper 25%
					3.60 (1.48–8.80)^†^	0.0049	GSE19234	Upper 25%
					1.93 (1.40–2.65)^†^	< 0.0001	TCGA	Upper 25%
*MITF*	[29]	200	OS	Poor	1.43 (1.09–1.87)^†^	0.0104	TCGA	Upper 50%
					3.46 (1.42–8.42)^†^	0.0063	GSE19234	Upper 50%
					3.33 (1.18–9.41)^†^	0.0230	GSE98394	Upper 50%
*KIF20A*	[30]	61	RFS	Poor	2.17 (1.12–4.20)^†^	0.0218	GSE22155	Upper 25%
					2.56 (1.20–5.47)^†^	0.0151	GSE50509	Upper 25%
					3.21 (1.26–8.20)^†^	0.0147	GSE98394	Upper 25%
					2.44 (1.02–5.83)^†^	0.0454	GSE19234	Upper 25%
*CTHRC1*	[31]	35	OS	Poor	3.41 (1.31–8.89)^†^	0.0122	GSE98394	Upper 25%
*TFAP2A*	[32]	Nearly 600	DSS	Poor	1.59 (1.03–2.47)^‡^	0.0379	GSE65904	Upper 25%
*ATF2*	[33]	544	OS	Poor	3.05 (1.56–5.97)^†^	0.0012	GSE22155	Upper 25%
*NCOA3*	[34]	343	RFS and DSS	Poor	1.79 (1.17–2.74)^‡^	0.0071	GSE65904	Upper 25%
*BCL2*	[35]	339	OS	Good	0.21 (0.04–0.97)^†^	0.0458	GSE22155	Upper 25%
*BIRC5*	[36]	50	DFS and OS	Poor	3.73 (1.44–9.67)^†^	0.0068	GSE98394	Upper 25%
*MCAM*	[37]	76	OS	Poor	4.66 (1.78–12.18)^†^	0.0017	GSE19234	Upper 25%
*PLAT*	[38]	214	DMFI and OS	Poor	2.24 (1.16–4.34)^†^	0.0164	GSE22155	Upper 25%
					3.88 (1.47–10.24)^†^	0.0063	GSE98394	Upper 25%
*NOS2*	[39]	132	DSS and OS	Poor	1.41 (1.07–1.85)^†^	0.0131	TCGA	Upper 50%
*CDKN1B*	[40]	383	DSS and OS	Poor	0.48 (0.24–0.95)^†^	0.0341	GSE22155	Upper 25%
					0.69 (0.50–0.95)^†^	0.0235	TCGA	Upper 25%
*BCL6*	[41]	88	6-year OS	Poor	0.57 (0.40–0.80)^†^	0.0011	TCGA	Upper 25%
*FXYD5*	[42]	115	OS	Poor	3.10 (1.24–7.76)^†^	0.0159	GSE19234	Upper 25%
*DDIT3*	[43]	106	OS	Good	5.74 (2.18–15.13)^†^	0.0004	GSE98394	Upper 25%
*MCAT*	[44]	1270	DFI and OS	Poor	5.75 (1.26–26.10)^†^	0.0236	GSE22155	Upper 25%
					4.51 (1.72–11.82)^†^	0.0021	GSE98394	Upper 25%
*CTNNB1*	[45]	202	DSS	Good	1.55 (1.02–2.37)^‡^	0.0412	GSE65904	Upper 25%
					1.75 (1.15–2.67)^‡^	0.0088	GSE65904	Upper 25%
*AKT1*	[46]	222	5-year DSS or OS	Poor	6.41 (2.39–17.23)^†^	0.0002	GSE98394	Upper 25%
					1.53 (1.13–2.06)^†^	0.0056	TCGA	Upper 25%
*RUNX3*	[47]	421	5-year OS	Good	3.75 (1.36–10.33)^†^	0.0107	GSE50509	Upper 25%
			5-year DSS		1.81 (1.18–2.76)^‡^	0.0062	GSE65904	Upper 25%
*BBC3*	[48]	158	5-year DSS or OS	Poor	3.62 (1.38–9.52)^†^	0.0092	GSE98394	Upper 25%
*MMP2*	[49]	157	DSS and RFS	Poor	1.41 (1.06–1.89)^‡^	0.0197	TCGA	Upper 50%
*SPP1*	[50]	345	RFS	Poor	9.42 (3.46–25.67)^†^	< 0.0001	GSE98394	Upper 25%
*TNC*	[51]	98	DFS	Poor	1.54 (1.01–2.34)^‡^	0.0434	GSE65904	Upper 25%
*CCNA2*	[52]	245	RFS	Poor	2.23 (1.02–4.88)^†^	0.0437	GSE50509	Upper 25%
*RGS1*	[53]	40	DSS	Poor	2.74 (1.03–7.24)^†^	0.0425	GSE98394	Upper 25%
					3.24 (1.31–8.00)^†^	0.0110	GSE19234	Upper 25%
					2.66 (1.07–6.65)^†^	0.0357	GSE19234	Upper 25%
*SPARC*	[54]	112	DFS	Poor	2.78 (1.21–6.34)^†^	0.0154	GSE50509	Upper 25%
*CXCR4*	[55]	71	DFS and OS	Poor	0.70 (0.51–0.97)^†^	0.0315	TCGA	Upper 25%
*RBM3*	[56]	246	OS	Good	NS	NS	–	–
*EPAS1*	[57]	46	DSS	Poor	3.51 (1.56–7.91)^†^	0.0024	GSE50509	Upper 25%

*NS* not significance, *RFS* recurrence-free survival, *DFS* disease-specific survival, *DFI* disease-free interval, *DMFI* distant metastasis-free interval

^†,‡^*HR* (95%CI) and Log-rank *P* value of overall survival (OS) and disease-specific survival (DSS)

The analysis of these reported prognostic biomarkers in *OSskcm* showed that the prognostic roles of 22 genes were consistent with previous findings, *RBM3* gene had no statistically significance on prognosis, and the other 7 genes (*KLK7*, *CXCR4*, *CDKN1B*, *BCL6*, *CTNNB1*, *RUNX3* and *DDIT3*) had opposite prognostic trends compared to literatures. The analysis results were presented in Table 2.

### Screening of new prognostic biomarkers for cutaneous melanoma

**Fig. 2:**
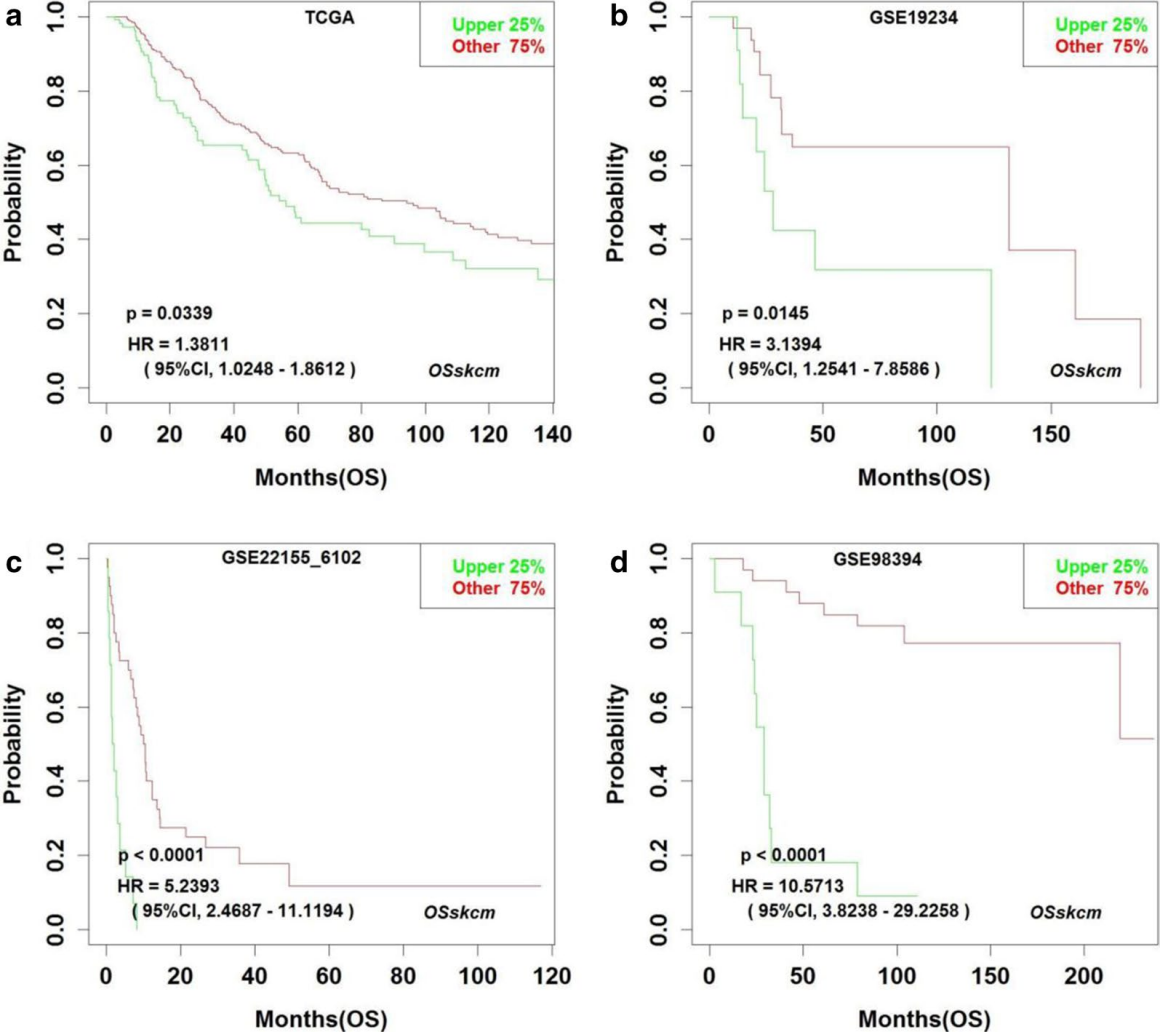
*SAE1* is identified as an unfavorable prognostic biomarker in *OSskcm*. Overall survival (OS) curve of cutaneous melanoma patients based on TCGA (**a**), GSE19234 (**b**), GSE22155 (**c**) and GSE98394 (**d**) data. Upper 25%: the SKCM cases with ranked top 25% higher expression level for the inputted gene; Other 75%: the SKCM cases with ranked bottom 75% lower expression level of the inputted gene

*OSskcm* can also be used to screen novel prognostic biomarkers for cutaneous melanoma, where OS, DSS, PFS, PFI and DMFS can be investigated. By *OSskcm*, we found that high expression of *SAE1* gene is associated with poor prognosis of cutaneous melanoma (Fig. 2), and the prognostic potency of *SAE1* gene has not been previously reported in cutaneous melanoma.

## Discussion

Due to the variant prognosis of cutaneous melanoma patients, the development of molecular prognosis biomarkers is significant. Here, we collected multiple large transcriptomic datasets to increase the statistical power for analyzing the association between the investigated marker and survival rate, and developed a freely accessible webserver *OSskcm* to estimate the prognostic value of any inputted gene in a large cohort of patients, by which KM survival curves as well as HR and log-rank *P* values could be outputted and presented. *OSskcm* is a webserver that can mutually validate prognostic biomarkers of cutaneous melanoma in multiple data sets. A total of 1085 patients of cutaneous melanoma with RNA-seq data from clinical tissues and clinical information were included in *OSskcm*. In addition, risk factors, including race, stage, gender, age and therapy type, can be selected for subgroup analysis. Clinical outcome data of OS, PFS, DSS, PFI, and DMFS was included in analysis.

We tested the performance of *OSskcm* using 30 previously reported cutaneous melanoma prognostic biomarkers. Among these, 22 genes were validated in *OSskcm*, but the prognostic significance of *RBM3*, *KLK7, CXCR4*, *CDKN1B*, *BCL6*, *CTNNB1*, *RUNX3* and *DDIT3* genes were inconsistent between literatures and *OSskcm*. It may be because the *OSskcm* utilizes mRNA expression data while all previously published biomarkers were studied based on the protein level. It is known that there is an inconsistency between the levels of mRNA and protein due to intracellular modifications, such as post-transcriptional regulation, protein translation and post-translational regulation. In addition, the prognostic significance of a protein may be determined by its subcellular localization. For example, loss of nuclear CDKN1B expression is correlated with a worse 5-year survival of primary melanoma patients in Kaplan–Meier analysis, but gain of cytoplasmic CDKN1B was associated with a poor 5-year survival of metastatic melanoma patients.

*KIF20A* and *RGS1* genes have been reported to play critical roles in the development and progression of cancer, and promote the proliferation, migration and invasion of cancer cells [58, 59]. In *OSskcm*, *KIF20A* and *RGS1* were found to be strongly associated with cutaneous melanoma prognosis. In addition, we found that *SAE1* could be a new prognostic biomarker in cutaneous melanoma. *SAE1* is dimeric SUMO Activating Enzyme E1, involves in SUMO conjugation [60]. Breast cancer patients with lower *SAE1* expression have been reported to have significantly lower instances of metastatic cancer and increased survival compared to those that express a higher level of *SAE1* [61]. Moreover, *SAE1* was reported to have the strongest synthetic lethal interactions with K-Ras and can be used to evaluate the aggressiveness of mutated K-Ras-dependent malignancies [62]. It will be interesting to further verify by experiments whether *SAE1* gene could be a new prognostic biomarker in cutaneous melanoma.

## Conclusion

In summary, by utilizing genome-wide microarray datasets and RNAseq datasets, we built a prognosis webserver, *OSskcm*, which offer a platform for biologists and clinicians to identify prognostic biomarkers for cutaneous melanoma. Additional more research regarding how to better translate our web server and web server derived biomarkers for practice from local to global health is required [63].

## Data Availability

The datasets used and analyzed during the current study are available from Gene Expression Omnibus (GEO; https://www.ncbi.nlm.nih.gov/geo/) and The Cancer Genome Atlas (TCGA; https://cancergenome.nih.gov/).

## Abbreviations

*OSskcm*: Online consensus Survival webserver for Skin Cutaneous Melanoma
GEO: Gene Expression Omnibus
TCGA: The Cancer Genome Atlas
OS: Overall survival
PFS: Progression-free survival
DSS: Disease-specific survival
PFI: Progression-free interval
DMFS: Distant metastasis-free survival
RFS: Recurrence-free survival
DFS: Disease-specific survival
DFI: Disease-free interval
DMFI: Distant metastasis-free interval
